# Supplementary material for: Leptospirosis occurs as frequently as malaria among adolescent and adult acute febrile patients in Hoima, Uganda: A prospective health facility-based study
Source: PLoS Negl Trop Dis. 2026 Oct 29;20(10):e0014772. doi: 10.1371/journal.pntd.0014772 (PMC13719026; doi:10.1371/journal.pntd.0014772)
Supplement: Supplementary file 1 [file pntd.0014772.s001.pdf]

S1 Table. STROBE Statement: Checklist of items that should be included in reports of cross-sectional studies

Leptospirosis occurs as frequently as malaria among adolescent and adult acute febrile patients in Hoima, Uganda: A prospective health facility-based study

Item No.	Item	Recommendation	Page No.
Title and Abstract			
1	**Title and abstract**	(a) Study design indicated in title: 'A prospective health facility-based study'. (b) Abstract provides balanced summary of objectives, design, setting, participants, key findings (prevalence 27.0%, risk factors, serovar distribution, qPCR-MAT concordance), and conclusions.	1-3
Introduction			
2	**Background / rationale**	Scientific background: leptospirosis underdiagnosis in sub-Saharan Africa, clinical overlap with malaria, prior Ugandan seroprevalence data (35% in 2016 Hoima study), livestock transmission evidence, exclusion from Uganda's national febrile illness algorithms.	5-6
3	**Objectives**	Specific objectives: determine prevalence of leptospirosis and co-infections with malaria and HIV, evaluate demographic, environmental, and occupational risk factors, generate evidence for national diagnostic and surveillance strategies.	6
Methods			
4	**Study design**	Key elements of study design: prospective health facility-based cross-sectional study with convalescent follow-up at 2-6 weeks.	6-7
5	**Setting**	Setting, locations, and relevant dates: Hoima Regional Referral Hospital (urban, serving approximately 4 million residents) and Kigorobya Health Centre IV (rural, subsistence farming and livestock keeping population), western Uganda, November 2023 to December 2024.	6-7
6	**Participants**	(a) Eligibility criteria: age 12 years or older, informed consent, fever 38.0 degrees Celsius or above or reported fever within preceding 14 days, no obvious focal infection. (b) Sources and methods of selection: dedicated research team screened at triage and inpatient wards. Numbers at each stage: 875 screened, 527 ineligible, 15 declined, 333 enrolled, 3 excluded (missing samples), 330 analysed.	6-7, 10
7	**Variables**	Outcome: confirmed acute leptospirosis (qPCR positivity, MAT seroconversion, fourfold titre rise, or single titre 1:800 or above). Exposures: demographic, occupational, animal contact, water source, flooding, vector exposure. Potential confounders: age, sex, and study site retained a priori.	8-9
8	**Data sources / measurement**	Data sources: REDCap electronic CRFs and standardised questionnaire for exposures. Diagnostic methods: LipL32 qPCR on whole blood and urine (TaqMan assay, cycle threshold below 41), MAT with 15-serovar panel on paired sera (initial screening at 1:50, serial twofold dilution to endpoint), malaria RDT, HIV testing, CBC, liver and kidney function, urinalysis. Extraction protocols, reagents, controls, and positivity criteria specified.	7-9
9	**Bias**	Efforts to address bias: facility-based design acknowledged to underrepresent mild community cases and pre-hospital deaths. Recall bias from self-reported single-timepoint exposures noted. Prior antibiotic use (73% received empiric doxycycline) reducing qPCR sensitivity and potentially blunting seroconversion and fourfold titre rises discussed. Loss to follow-up characterised by age and sex.	20-21
10	**Study size**	Sample size rationale: minimum 175 required based on prior 6% prevalence estimate (epitools, 95% confidence, 5% precision); enrolled 330 providing 80% power to detect odds ratio of 2.5 or higher.	7
11	**Quantitative variables**	Age categorised into quintiles (12-17, 18-21, 22-28, 29-37, 38-65) for regression. Symptom duration reported as median with IQR. MAT titres categorised by seroconversion thresholds. Laboratory parameters categorised as low, normal, or high using standard clinical reference ranges. Prevalence reported with 95% CIs.	9-10, Appendix Tables 2-4

12	**Statistical methods**	(a) Univariable and multivariable logistic regression with change-in-estimate confounder selection and likelihood ratio testing (p 0.05 or below). (b) Subgroup comparison by study site. (c) Loss to follow-up: 49/333 (14.7%) characterised as younger but comparable in sex. (d) Not applicable (cross-sectional). (e) Sensitivity analyses: conservative vs lenient case definitions applied throughout.	9
Results			
13	**Participants**	(a) Numbers at each stage: 875 screened, 527 ineligible, 15 declined, 333 enrolled, 3 excluded for missing samples, 330 analysed. Participant flow diagram provided (Figure 1). (b) Non-participation: 15 declined consent. (c) Flow diagram included.	10-11
14	**Descriptive data**	(a) Participant characteristics by study site: Hoima-RRH (n=118) and Kigorobya-HCIV (n=212). Demographics, exposure variables, clinical features, and laboratory parameters described. (b) Missing data: 49 lost to convalescent follow-up characterised. (c) Follow-up: paired sera from 284/333 (85.3%).	10-16
15	**Outcome data**	Number of outcome events reported: 89 conservative cases (27.0%), 108 lenient cases (32.7%), 30 qPCR positive (9.1%), seroprevalence 35.8%. Results by site: Kigorobya-HCIV 34.9% vs Hoima-RRH 12.7%. Malaria 30.3%, HIV 7.6%, co-infections 8.8% and 1.5%.	10-13, Table 1
16	**Main results**	(a) Unadjusted and adjusted estimates with CIs and p-values: skinning animals aOR 5.19 (95% CI 1.40-21.16, p=0.015), mosquito exposure aOR 2.31 (95% CI 1.17-4.70, p=0.018). (b) Confounders: age, sex, study site retained a priori; additional covariates selected by change-in-estimate and likelihood ratio test. (c) Continuous variables categorised as described in item 11.	14-15, Figure 3, Appendix Table 2
17	**Other analyses**	Clinical features by univariable regression (justified as downstream consequences; Appendix Table 3). Laboratory parameters by univariable regression (Appendix Table 4). Serovar distribution (Appendix Table 5). MAT-qPCR agreement (kappa -0.26). Temporal distribution of cases plotted against rainfall (Figure 2). Conservative vs lenient case definitions compared.	15-17, Figures 2-3, Appendix Tables 2-5
Discussion			
18	**Key results**	Key results summarised: conservative prevalence 27.0%, comparable to malaria at 30.3%. Skinning animals and mosquito exposure as independent risk factors. Sv Bataviae dominant, first report in Uganda. Poor qPCR-MAT concordance (kappa -0.26) confirming need for combined diagnostics.	17
19	**Limitations**	Limitations discussed: facility-based design underrepresenting mild and fatal cases; single district limiting generalisability; exclusion of children under 12; loss to follow-up biased toward younger adults; prior antibiotic use reducing qPCR sensitivity; empiric doxycycline blunting seroconversion; self-reported exposure recall bias; sparse data for some variables; stage-dependent diagnostic sensitivity; MAT cross-reactivity; absence of latent-class modelling.	20-21
20	**Interpretation**	Cautious interpretation in context of prior evidence: higher prevalence attributed to composite diagnostic approach and catchment ecology. Site differences explained by mediation through rural exposures. Comparisons with prior Ugandan, East African, and multi-country AFI estimates. Negative kappa interpreted as stage-dependent temporal discordance.	17-20
21	**Generalisability**	Generalisability discussed: findings may underrepresent mild community cases and pre-hospital deaths; limited to Hoima district; children excluded. Multisite studies across Uganda and East Africa recommended.	20-21
Other Information			
22	**Funding**	Funding: Swiss National Science Foundation SPIRIT project (IZSTZ0_190156). Role of funding source: funder had no role in study design, data collection, data analysis, data interpretation, or writing of the report.	3, 9-10, 22
