# Supplementary material for: Leptospirosis occurs as frequently as malaria among adolescent and adult acute febrile patients in Hoima, Uganda: A prospective health facility-based study
Source: PLoS Negl Trop Dis. 2026 Oct 29;20(10):e0014772. doi: 10.1371/journal.pntd.0014772 (PMC13719026; doi:10.1371/journal.pntd.0014772)
Supplement: Supplementary file 3 [file pntd.0014772.s003.pdf]

S2 Text: Results

Loss-to-Follow up distributions

Among the 49 participants who were lost to follow-up, 35 (71.4%) were female and 14 (28.6%) were male. In this group, 4 (8.2%) were 12–17 years old, 13 (26.5%) were 18–21 years, 17 (34.7%) were 22–28 years, 7 (14.3%) were 29–37 years, and 8 (16.3%) were 38–65 years. Among the 284 participants who completed follow-up, 204 (71.8%) were female and 80 (28.2%) were male; 11 (3.9%) were 12–17 years old, 66 (23.2%) were 18–21 years, 62 (21.8%) were 22–28 years, 66 (23.2%) were 29–37 years, and 79 (27.8%) were 38–65 years. The gender distribution within the loss-to-follow-up group approximated that in the group that completed follow-up.
