# Supplementary material for: Leptospirosis occurs as frequently as malaria among adolescent and adult acute febrile patients in Hoima, Uganda: A prospective health facility-based study
Source: PLoS Negl Trop Dis. 2026 Oct 29;20(10):e0014772. doi: 10.1371/journal.pntd.0014772 (PMC13719026; doi:10.1371/journal.pntd.0014772)
Supplement: Supplementary file 4 [file pntd.0014772.s004.pdf]

S2 Table. Risk Factors Associated with Leptospirosis in Febrile Patients (n = 330), Hoima, Uganda

Risk Factor	Sub-group	n (%)	Leptospirosis Cases (%)	Crude OR	95% CI	p-value (Crude)	Adjusted OR	95% CI (Adj)	p-value (Adj)
Age (years)	12-17	15(4.5)	5(5.6)	Ref			Ref		
	18-21	85 (25.8)	31 (34.8)	0.69	0.22, 2.43	0.54	0.53	0.12, 2.29	0.382
	22-28	78 (23.6)	26 (29.2)	0.55	0.17, 1.95	0.327	0.39	0.08, 1.82	0.225
	29-37	73 (22.1)	26 (29.2)	0.81	0.25, 2.85	0.724	0.50	0.11, 2.34	0.371
	38-65	79 (23.9)	20 (22.5)	0.88	0.28, 3.06	0.832	0.46	0.1, 2.08	0.304
Sex	Female	238 (72.1)	71 (79.8)	Ref			Ref		
	Male	92 (27.9)	18 (20.2)	0.57	0.31, 1.01	0.061	0.64	0.33, 1.22	0.187
Study site	Hoima-RRH	118 (35.8)	15 (16.8)	Ref			Ref		
	Kigoroby a-HCIV	212 (64.2)	74 (83.1)	3.68	2.05, 7	<0.001	1.67	0.62, 4.62	0.313

Risk Factor	Sub-group	n (%)	Leptospirosis Cases (%)	Crude OR	95% CI	p-value (Crude)	Adjusted OR	95% CI (Adj)	p-value (Adj)
Level of education	No answer	1 (0.3)	1 (1.1)						
	None	26 (7.9)	6 (6.7)		***				
	Primary	191 (57.9)	51 (57.3)		***				
	Secondary	98 (29.7)	29 (32.6)		***				
	Tertiary	14 (4.2)	2 (2.2)		***				
Occupation	No	73 (22.1)	11 (12.4)	Ref			Ref		
	Yes	257 (77.9)	78 (87.6)	2.46	1.27, 5.15	0.011	2.26	0.81, 6.73	0.128
Use of protective equipment at work	Always	23 (7.0)	7 (7.9)	Ref					
	Never	206 (62.4)	56 (62.9)	0.85	0.34, 2.32	0.741			
	Sometimes	101 (30.6)	26 (29.2)	0.79	0.3, 2.25	0.646			
Student	No	328 (99.4)	89 (100)						
	Yes	2 (0.6)	0 (0.0)		***				
Professional	No	321 (97.3)	86 (96.6)	Ref					
	Yes	9 (2.7)	3 (3.4)	1.37	0.28, 5.3	0.664			
Business	No	289 (87.6)	82 (92.1)	Ref					
	Yes	41 (12.4)	7 (7.9)	0.52	0.2, 1.15	0.133			
Agricultural farmer	No	145 (43.9)	26 (29.2)	Ref			Ref		
	Yes	185 (56.1)	63 (70.8)	2.36	1.42, 4.04	0.001	0.96	0.41, 2.23	0.914
Livestock farmer	No	220 (66.7)	52 (58.4)	Ref			Ref		
	Yes	110 (33.3)	37 (41.6)	1.64	0.99, 2.71	0.055	0.8	0.41, 1.55	0.512
Herding	No	174 (52.7)	43 (48.3)	Ref			Ref		
	Yes	156 (47.3)	46 (51.7)	1.27	0.78, 2.08	0.33	1.20	0.50, 2.85	0.673
Milking	No	324 (98.2)	87 (97.7)	Ref					
	Yes	6 (1.8)	2 (2.2)	1.36	0.19, 7.11	0.724			
Slaughtering	No	296 (89.7)	76 (85.4)	Ref			Ref		
	Yes	34 (10.3)	13 (14.6)	1.79	0.84, 3.71	0.122	1.44	0.38, 5.10	0.577
Skinning animals	No	317 (96.1)	82 (92.1)	Ref					
	Yes	13 (3.9)	7 (7.9)	3.34	1.08, 10.66	0.034	5.19	1.4, 21.16	0.015
Butchering	No	313 (94.8)	85 (95.5)	Ref			Ref		

Risk Factor	Sub-group	n (%)	Leptospirosis Cases (%)	Crude OR	95% CI	p-value (Crude)	Adjusted OR	95% CI (Adj)	p-value (Adj)
	Yes	17 (5.2)	4 (4.5)	0.82	0.23, 2.41	0.743	0.58	0.08, 3.04	0.550
Grooming of animals	No	208 (63.0)	54 (60.7)	Ref			Ref		
	Yes	122 (37.0)	35 (39.3)	1.15	0.69, 1.89	0.59	0.62	0.25, 1.53	0.303
Taking care of sick animals	No	318 (96.4)	86 (96.6)	Ref					
	Yes	12 (3.6)	3 (3.4)	0.9	0.2, 3.1	0.876			
Fishing	No	322 (97.6)	87 (97.7)	Ref					
	Yes	8 (2.4)	2 (2.2)	0.9	0.13, 3.99	0.899			
Heavy rains at home or workplace	No	34 (10.3)	13 (14.6)	Ref			Ref		
	Yes	296 (89.7)	76 (85.4)	0.56	0.27, 1.2	0.122	0.41	0.15, 1.07	0.068
Flooding Near Home/Workplace	No	239 (72.4)	80 (89.9)	Ref			Ref		
	Yes	91 (27.6)	9 (10.1)	0.22	0.1, 0.44	<0.001	0.45	0.16, 1.15	0.102
Standing in shallow water	No	295 (89.4)	83 (93.3)	Ref					
	Yes	35 (10.6)	6 (6.7)	0.53	0.19, 1.24	0.172			
Taking Bottled water	No	130 (39.4)	19 (21.3)	Ref			Ref		
	Yes	200 (60.6)	70 (78.7)	3.15	1.82, 5.67	<0.001	1.46	0.7, 3.13	0.321
Using Surface water	No	279 (84.5)	78 (87.6)	Ref			Ref		
	Yes	51 (15.5)	11 (12.4)	0.71	0.33, 1.41	0.346	0.74	0.28, 1.88	0.542
Using Borehole	No	89 (27.0)	21 (23.6)	Ref					
	Yes	241 (73.0)	68 (76.4)	1.27	0.73, 2.27	0.402			
Using Well water	No	190 (57.6)	48 (53.9)	Ref					
	Yes	140 (42.4)	41 (46.1)	1.23	0.75, 2	0.416			
Using Public tap	No	272 (82.4)	75 (84.3)	Ref					
	Yes	58 (17.6)	14 (15.7)	0.84	0.42, 1.58	0.593			
Using Home piped water	No	289 (87.6)	78 (87.6)	Ref					
	Yes	41 (12.4)	11 (12.4)	0.99	0.46, 2.02	0.983			
Eating Goat meat	No	192 (58.2)	66 (74.2)	Ref			Ref		
	Yes	138 (41.8)	23 (25.8)	0.38	0.22, 0.65	<0.001	0.97	0.44, 2.1	0.933
Contact with animals within the last 2 weeks	No	159 (48.2)	42 (47.2)	Ref					
	Yes	171 (51.8)	47 (52.8)	1.06	0.65, 1.72	0.827			
Contact with Cattle	No	301 (91.2)	86 (96.6)	Ref			Ref		
	Yes	29 (8.8)	3 (3.4)	0.29	0.07, 0.85	0.046	0.26	0.05, 0.9	0.056

Risk Factor	Sub-group	n (%)	Leptospirosis Cases (%)	Crude OR	95% CI	p-value (Crude)	Adjusted OR	95% CI (Adj)	p-value (Adj)
Contact with Dogs	No	280 (84.8)	77 (86.5)	Ref					
	Yes	50 (15.2)	12 (13.5)	0.83	0.4, 1.63	0.608			
Contact with Cats	No	282 (85.5)	79 (88.8)	Ref					
	Yes	48 (14.5)	10 (11.2)	0.68	0.31, 1.37	0.302			
Contact with Chicken	No	143 (43.3)	42 (47.2)	Ref					
	Yes	187 (56.7)	47 (52.8)	0.81	0.49, 1.32	0.391			
Contact with Pigs	No	228 (69.1)	64 (71.9)	Ref					
	Yes	102 (30.9)	25 (28.1)	0.83	0.48, 1.41	0.501			
Contact with Goats	No	243 (73.6)	71 (79.8)	Ref					
	Yes	87 (26.4)	18 (20.2)	0.63	0.34, 1.12	0.126			
Contact with Poultry other than chickens	No	165 (50.0)	38 (42.7)	Ref					
	Yes	165 (50.0)	51 (57.3)	1.5	0.92, 2.45	0.108			
Wild animals Near Home/Workplace	No	125 (37.9)	21 (23.6)	Ref			Ref		
	Yes	205 (62.1)	68 (76.4)	2.46	1.44, 4.35	0.001	0.64	0.14, 2.63	0.543
Exposure to Mosquitoes	No	150 (45.5)	23 (25.8)	Ref			Ref		
	Yes	180 (54.5)	66 (74.2)	3.2	1.89, 5.56	<0.001	2.31	1.17, 4.7	0.018
Exposure to Rats	No	139 (42.1)	26 (29.2)	Ref			Ref		
	Yes	191 (57.9)	63 (70.8)	2.14	1.28, 3.65	0.004	0.84	0.24, 3.23	0.790
Exposure to Bats	No	187 (56.7)	39 (43.8)	Ref			Ref		
	Yes	143 (43.3)	50 (56.2)	2.04	1.25, 3.35	0.004	1.39	0.68, 2.9	0.368
Exposure to Monkeys	No	221 (67.0)	50 (56.2)	Ref			Ref		
	Yes	109 (33.0)	39 (43.8)	1.9	1.15, 3.15	0.012	1.23	0.64, 2.39	0.538
Exposure to Rodents	No	158 (47.9)	36 (40.4)	Ref			Ref		
	Yes	172 (52.1)	53 (59.6)	1.51	0.92, 2.48	0.102	0.74	0.39, 1.42	0.366
Exposure to Baboons	No	317 (96.1)	83 (93.3)	Ref					
	Yes	13 (3.9)	6 (6.7)	2.42	0.76, 7.48	0.122			
Exposure to Chimpanzees	No	326 (98.8)	88 (98.9)	Ref					
	Yes	4 (1.2)	1 (1.1)	0.9	0.04, 7.15	0.929			
Consuming Beef	No	116 (35.2)	31 (34.8)	Ref					
	Yes	214 (64.8)	58 (65.2)	1.02	0.61, 1.71	0.941			
Consuming Pork	No	185 (56.1)	57 (64.0)	Ref			Ref		

Risk Factor	Sub-group	n (%)	Leptospirosis Cases (%)	Crude OR	95% CI	p-value (Crude)	Adjusted OR	95% CI (Adj)	p-value (Adj)
	Yes	145 (43.9)	32 (36.0)	0.64	0.38, 1.04	0.077	1.17	0.64, 2.12	0.612
Consuming Raw milk	No	307 (93.0)	86 (96.6)	Ref					
	Yes	23 (7.0)	3 (3.4)	0.38	0.09, 1.16	0.132			
Consuming Boiled milk	No	166 (50.3)	49 (55.1)	Ref					
	Yes	164 (49.7)	40 (44.9)	0.77	0.47, 1.25	0.294			
Consuming Animal blood	No	315 (95.5)	86 (96.6)	Ref					
	Yes	15 (4.5)	3 (3.4)	0.67	0.15, 2.16	0.536			
Consuming Fermented milk	No	284 (86.1)	78 (87.6)	Ref					
	Yes	46 (13.9)	11 (12.4)	0.83	0.38, 1.67	0.615			

Crude odds ratios (OR) are from univariable logistic regression. Adjusted odds ratios (aOR) are from multivariable logistic regression including variables with $p \leq 0.20$ in univariable analysis and biologically plausible exposures; age, sex, and study site were retained as a priori confounders regardless of statistical significance. Leptospirosis was defined using the conservative case definition. Certain crude associations reflect site-level confounding rather than causal relationships: the elevated crude OR for bottled water use (3.15) and the apparently protective crude OR for flooding near home or workplace (0.22) both attenuate and lose significance after multivariable adjustment, consistent with differential exposure distributions between the higher-prevalence rural site (Kigorobya-HCIV) and the lower-prevalence urban site (Hoima-RRH). Variables with sparse data (expected cell count <5 or zero cases in one category) were excluded from regression modelling and are marked with asterisks. Ref = reference category. CI = confidence interval. RRH = regional referral hospital. HCIV = health centre IV.
