# Supplementary material for: Leptospirosis occurs as frequently as malaria among adolescent and adult acute febrile patients in Hoima, Uganda: A prospective health facility-based study
Source: PLoS Negl Trop Dis. 2026 Oct 29;20(10):e0014772. doi: 10.1371/journal.pntd.0014772 (PMC13719026; doi:10.1371/journal.pntd.0014772)
Supplement: Supplementary file 5 [file pntd.0014772.s005.pdf]

S3 Table. Clinical symptoms, signs, and pre-enrolment medication use among 330 acute undifferentiated fever patients by leptospirosis case status, Hoima, Uganda, November 2023 to December 2024

Clinical Symptom or sign	Sub-group	n (%)	Leptospirosis Cases (%)	Crude OR	95% CI	p-value
Days symptomatic	1	4 (1.2)	0 (0.0)			
	2	44 (13.3)	10 (11.2)			
	3	54 (16.4)	11 (12.4)			
	4	36 (10.9)	6 (6.7)			
	5	20 (6.1)	9 (10.1)			
	6	11 (3.3)	1 (1.1)			
	7	70 (21.2)	25 (28.1)			
	8	11 (3.3)	4 (4.5)			
	9	3 (0.9)	1 (1.1)			
	10	6 (1.8)	2 (2.2)			
	11	3 (0.9)	2 (2.2)			
	12	3 (0.9)	0 (0.0)			
	13	40 (12.1)	12 (13.5)			
	14	24 (7.3)	6 (6.7)			
	17	1 (0.3)	0 (0.0)			
General appearance	Fair condition	314 (95.2)	85 (95.5)	Ref		
	Sick looking	16 (4.8)	4 (4.5)	0.9	0.25, 2.66	0.856
Pallor	No	321 (97.3)	89 (100)	Ref		
	Yes	9 (2.7)	0 (0.0%)		***	
Yellowing of the eyes	No	319 (96.7)	85 (95.5)	Ref		
	Yes	11 (3.3)	4 (4.5)	1.57	0.4, 5.34	0.479

Clinical Symptom or sign	Sub-group	n (%)	Leptospirosis Cases (%)	Crude OR	95% CI	p-value
Fatigue	No	96 (29.1)	21 (23.6)	Ref		
	Yes	234 (70.9)	68 (76.4)	1.46	0.85, 2.61	0.183
Chills	No	313 (94.8)	77 (86.5)	Ref		
	Yes	17 (5.2)	12 (13.5)	7.36	2.64, 23.73	<0.001
Swelling of the eyes	No	328 (99.4)	87 (97.8)	Ref		
	Yes	2 (0.6)	2 (2.2)		***	
Lymphadenopathy	No	327 (99.1)	89 (100)	Ref		
	Yes	3 (0.9)	0 (0.0)		***	
Running nose	No	211 (63.9)	63 (70.8)	Ref		
	Yes	119 (36.1)	26 (29.2)	0.66	0.38, 1.1	0.117
Sore throat	No	284 (86.1)	80 (89.9)	Ref		
	Yes	46 (13.9)	9 (10.1)	0.62	0.27, 1.29	0.226
Chest pain	No	305 (92.4)	79 (88.8)	Ref		
	Yes	25 (7.6)	10 (11.2)	1.91	0.8, 4.38	0.132
Cough	No	284 (86.1)	70 (78.7)	Ref		
	Yes	46 (13.9)	19 (21.3)	2.15	1.12, 4.09	0.02
Difficulty in breathing	No	324 (98.2)	86 (96.6)	Ref		
	Yes	6 (1.8)	3 (3.4)	2.77	0.5, 15.2	0.218
Heart murmur	No	326 (98.8)	87 (97.8)	Ref		
	Yes	4 (1.2)	2 (2.2)	2.75	0.33, 23.18	0.316
Hypertension	No	318 (96.4)	85 (95.5)	Ref		
	Yes	12 (3.6)	4 (4.5)	1.37	0.36, 4.47	0.614
Abdominal pain	No	268 (81.2)	66 (74.2)	Ref		
	Yes	62 (18.8)	23 (25.8)	1.8	0.99, 3.22	0.048
Diarrhoea	No	326 (98.8)	86 (96.6)	Ref		
	Yes	4 (1.2)	3 (3.4)	8.37	1.06, 170.49	0.067
Abdominal distension	No	325 (98.5)	87 (97.8)	Ref		
	Yes	5 (1.5)	2 (2.2)	1.82	0.24, 11.18	0.514
Hepatomegaly	No	329 (99.7)	88 (98.9)	Ref		
	Yes	1 (0.3)	1 (1.1)		***	
Jaundice	No	320 (97.0)	88 (98.9)	Ref		
	Yes	10 (3.0)	1 (1.1)	0.29	0.02, 1.59	0.247
Nausea	No	232 (70.3)	59 (66.3)	Ref		
	Yes	98 (29.7)	30 (33.7)	1.29	0.76, 2.17	0.333
Reduced appetite	No	130 (39.4)	26 (29.2)	Ref		
	Yes	200 (60.6)	63 (70.8)	1.84	1.1, 3.14	0.022
Splenomegaly	No	328 (99.4)	88 (98.9)	Ref		
	Yes	2 (0.6)	1 (1.1)	2.73	0.11, 69.46	0.48
Vomiting	No	321 (97.3)	87 (97.8)	Ref		

Clinical Symptom or sign	Sub-group	n (%)	Leptospirosis Cases (%)	Crude OR	95% CI	p-value
	Yes	9 (2.7)	2 (2.2)	0.77	0.11, 3.25	0.746
Diabetes	No	327 (99.1)	88 (98.9)	Ref		
	Yes	3 (0.9)	1 (1.1)	1.36	0.06, 14.35	0.804
Dysuria	No	297 (90.0)	77 (86.5)	Ref		
	Yes	33 (10.0)	12 (13.5)	1.63	0.75, 3.43	0.203
Urethral discharge	No	258 (78.2)	63 (70.8)	Ref		
	Yes	72 (21.8)	26 (29.2)	1.75	0.99, 3.04	0.05
Urine frequency	No	263 (79.7)	65 (73.0)	Ref		
	Yes	67 (20.3)	24 (27.0)	1.7	0.95, 3	0.069
Renal disease	No	329 (99.7)	89 (100)	Ref		
	Yes	1 (0.3)	0 (0.0)		***	
Confusion	No	326 (98.8)	86 (96.6)	Ref		
	Yes	4 (1.2)	3 (3.4)	8.37	1.06, 170.49	0.067
Convulsions	No	327 (99.1)	87 (97.8)	Ref		
	Yes	3 (0.9)	2 (2.2)	5.52	0.52, 119.6	0.165
Headache	No	269 (81.5)	64 (71.9)	Ref		
	Yes	61 (18.5)	25 (28.1)	2.22	1.23, 3.97	0.007
Muscle/joint pain	No	290 (87.9)	74 (83.1)	Ref		
	Yes	40 (12.1)	15 (16.9)	1.75	0.86, 3.47	0.113
Tenderness	No	249 (75.5)	58 (65.2)	Ref		
	Yes	81 (24.5)	31 (34.8)	2.04	1.19, 3.48	0.009
Skin rash	No	328 (99.4)	88 (98.9)	Ref		
	Yes	2 (0.6)	1 (1.1)	2.73	0.11, 69.46	0.48
Any abnormal bleeding	No	329 (99.7)	88 (98.9)	Ref		
	Yes	1 (0.3)	1 (1.1)		***	
Diagnosed with an infection within the last year	No	168 (50.9)	34 (38.2)	Ref		
	Yes	162 (49.1)	55 (61.8)	2.03	1.24, 3.35	0.005
Took antiretroviral ART within the last 2 weeks	No	314 (95.2)	84 (94.4)	Ref		
	Yes	16 (4.8)	5 (5.6)	1.25	0.38, 3.53	0.693
Took antimalarials within the last 2 weeks	No	277 (83.9)	74 (83.1)	Ref		
	Yes	53 (16.1)	15 (16.9)	1.08	0.55, 2.05	0.811
Took antibiotics within the last 2 weeks	No	284 (86.1)	75 (84.3)	Ref		
	Yes	46 (13.9)	14 (15.7)	1.22	0.6, 2.37	0.569
Did not take any medicine within the last 2 weeks	No	179 (54.2)	36 (40.4)	Ref		
	Yes	151 (45.8)	53 (59.6)	2.15	1.31, 3.55	0.002

Crude odds ratios (OR) are from univariable logistic regression comparing confirmed leptospirosis cases (conservative definition; n=89) with febrile non-cases (n=241). Multivariable adjustment was not performed for clinical features, as these represent downstream consequences of infection rather than independent risk factors, and multivariable modelling of correlated disease manifestations risks overadjustment. Days symptomatic are reported as counts without regression modelling (median 7 days, IQR 4-9). Variables with sparse data (expected cell count <5 or zero cases in one category) were excluded from regression modelling and are marked with asterisks. All comparisons are against febrile non-cases rather than healthy controls, reflecting the clinical diagnostic context and limiting discriminatory value for parameters common to multiple febrile aetiologies. Ref = reference category. CI = confidence interval.
