# Supplementary material for: Leptospirosis occurs as frequently as malaria among adolescent and adult acute febrile patients in Hoima, Uganda: A prospective health facility-based study
Source: PLoS Negl Trop Dis. 2026 Oct 29;20(10):e0014772. doi: 10.1371/journal.pntd.0014772 (PMC13719026; doi:10.1371/journal.pntd.0014772)
Supplement: Supplementary file 6 [file pntd.0014772.s006.pdf]

S4 Table. Laboratory parameters by leptospirosis case status among 330 acute undifferentiated fever patients at two health facilities in Hoima, Uganda, November 2023 to December 2024

Laboratory Parameter	Parameter Normal ranges	Sub-group	n (%)	Leptospirosis Cases (%)	Crude OR	95% CI	p-value
ALT (U/L)	Female (10-35) Male (10-50)	Low	30 (9.1)	9 (10.1)	1.16	0.48, 2.58	0.727
		Normal	283 (85.8)	78 (87.6)	Ref		
		High	17 (5.2)	2 (2.2)	0.51	0.12, 1.61	0.304
AST (U/L)	Female (10-35) Male (10-50)	Normal	288 (87.3)	82 (92.1)	Ref		
		High	42 (12.7)	7 (7.9)	0.56	0.23, 1.20	0.161
Haemoglobin (g/dL)	12-16	Low	40 (12.1)	11 (12.4)	0.92	0.42, 1.85	0.820
		Normal	236 (71.5)	69 (77.5)	Ref		
		High	54 (16.4)	9 (10.1)	**0.47**	0.20, 0.96	**0.051**
Platelets (x10^3/uL)	100-300	Low	22 (6.7)	5 (5.6)	0.64	0.2, 2	0.44
		Normal	250 (75.8)	66 (74.2)	Ref		
		High	57 (17.3)	18 (20.2)	0.46	0.26, 0.81	0.739
White blood cell count (cells/uL)	4-10	Low	66 (20.0)	16 (18.0)	0.74	0.36, 1.42	0.379
		Normal	247 (74.8)	68 (76.4)	Ref		
		High	17 (5.2)	5 (5.6)	1.04	0.32, 2.91	0.941
Neutrophils (cells/uL)	2-7	Low	95 (28.8)	27 (30.3)	1.12	0.65, 1.89	0.673
		Normal	225 (68.2)	59 (66.3)	Ref		
		High	10 (3.0)	3 (3.4)	1.17	0.24, 4.34	0.828
Lymphocytes (cells/uL)	0.8-4	Low	85 (25.8)	**8 (9.0)**	0.20	0.09, 0.41	**<0.0001**
		Normal	237 (71.8)	79 (88.8)	Ref		
		High	8 (2.4)	2 (2.2)	0.64	0.09, 2.86	0.593
Eosinophils (cells/uL)	0.02-0.5	Low	235 (71.2)	70 (78.7)	1.77	1.00, 3.25	**0.06**
		Normal	90 (27.3)	18 (20.2)	Ref		
		High	5 (1.5)	1 (1.1)	1.00	0.05, 7.29	1.00
Creatinine (umol/L)	44-80	Low	33 (10.0)	13 (14.6)	2.04	0.95, 4.28	0.062
		Normal	238 (72.1)	62 (69.7)	Ref		
		High	59 (17.9)	14 (15.7)	0.86	0.43, 1.64	0.657
Number of pus cells in urine (cells)	0-5	Normal	178 (53.9)	43 (48.3)	Ref		
		High	152 (46.1)	46 (51.7)	1.39	0.86, 2.25	0.181
Number of Red cells in urine (cells)	0-3	Normal	319 (96.7)	87 (97.8)	Ref		
		High	11 (3.3)	2 (2.2)	0.87	0.19, 2.99	0.836

Laboratory Parameter	Parameter Normal ranges	Sub-group	n (%)	Leptospirosis Cases (%)	Crude OR	95% CI	p-value
CRP (mg/L)	0-5	Normal	197 (59.7)	49 (55.1)	Ref		
		High	133 (40.3)	40 (44.9)	1.34	0.82, 2.17	0.241
HIV infection		No	305 (92.4)	84 (94.4)	Ref		
		Yes	25 (7.6)	5 (5.6)	0.77	0.27, 1.88	0.590
Malaria infection		No	230 (69.7)	60 (67.4)	Ref		
		Yes	100 (30.3)	29 (32.6)	1.16	0.69, 1.93	0.576

Abbreviations: ALT – Alanine aminotransferase, **AST** – Aspartate aminotransferase, **CRP -** C reactive protein

Crude odds ratios (OR) are from univariable logistic regression comparing confirmed leptospirosis cases (conservative definition; n=89) with febrile non-cases (n=241). Laboratory values were categorised as low, normal, or high based on standard clinical reference ranges shown in the second column. Multivariable adjustment was not performed, consistent with the approach for clinical features (Table 3). All comparisons are against febrile non-cases rather than healthy controls, which limits discriminatory value for parameters affected by multiple febrile aetiologies. Lymphopenia was the only parameter significantly associated with leptospirosis case status, with markedly lower frequency among cases than non-cases (9.0% vs 31.9%; p<0.0001). ALT = alanine aminotransferase. AST = aspartate aminotransferase. CRP = C-reactive protein. Ref = reference category. CI = confidence interval.
