# Supplementary material for: Leptospirosis occurs as frequently as malaria among adolescent and adult acute febrile patients in Hoima, Uganda: A prospective health facility-based study
Source: PLoS Negl Trop Dis. 2026 Oct 29;20(10):e0014772. doi: 10.1371/journal.pntd.0014772 (PMC13719026; doi:10.1371/journal.pntd.0014772)
Supplement: Supplementary file 7 [file pntd.0014772.s007.pdf]

S5 Table. Distribution and seroprevalence of *Leptospira* serovars among 330 acute undifferentiated fever patients at two health facilities in Hoima, Uganda, November 2023 to December 2024

Species	Serogroup	Serovar	Strain	N Seropositive[1] Seroprev (%)	95% CI Seroprev	N cases by serology[2], Prev (%)	95% CI Prev
L. interrogans	Icterohaemorrhagiae	Ict	RGA	1 (0.3)	0.1-1.7	1 (0.3)	0.1-1.7
	Pomona	Pom	Pomona	0 (0.0)	0.0-1.2	0 (0.0)	0.0-1.2
	Hebdomadis	Heb	Hebdomadis	3 (0.9)	0.3-2.6	1 (0.3)	0.1-1.7
	Australis	Aus	Ballico	21 (6.4)	4.2-9.5	15 (4.5)	2.8-7.4
	Canicola	Can	Strain Hond Utrecht IV	8 (.4)	1.2-4.7	5 (1.5)	0.6-3.5
	Bataviae	Bat	Swart	79 (23.9)	19.7-28.8	60 (18.2)	14.4-22.7
	Djasiman	Djs	Djasiman	1 (0.3)	0.1-1.7	1 (0.3)	0.1-1.7
L. borgpetersenii	Sejroe	Sej	M84	0 (0.0)	0.0-1.2	0 (0.0)	0.0-1.2
		Har	Sponselee	1 (0.3)	0.1-1.7	0 (0.0)	0.0-1.2
	Ballum	Ken	Njenga	19 (5.8)	3.7-8.8	8 (2.4)	1.2-4.7
	Pyrogenes	Nig	Vom	3 (0.9)	0.3-2.6	3 (0.9)	0.3-2.6
	Tarassovi	Tar	Perepelitsin	17 (5.2)	3.2-8.1	11 (3.3)	1.9-5.9
L. kirschneri	Autumnalis	But	Butembo	0 (0.0)	0.0-1.2	0 (0.0)	0.0-1.2
	Grippotyphosa	Gri	Duyster	1 (0.3)	0.1-1.7	1 (0.3)	0.1-1.7

Abbreviations: Sej = Sejroe, Ict = Icterohaemorrhagiae, Pom = Pomona, But = Butembo, Gri = Grippotyphosa, Heb = Hebdomadis, Ken = Kenya, Nig = Nigeria, Cel = Celledoni, Aus = Australis, Tar = Tarassovi, Can = Canicola, Har = Hardjo type Bovis, Bat = Bataviae, Djs = Djasiman., Seroprev = seroprevalence, Prev = prevalence. [1] Total number of participants with samples positive to MAT (Acute and/or convalescent) at 1:100 titre. [2] Total number of participants being serological cases: seroconversion to ≥1:200, fourfold rise in titre between paired sera, or single titre ≥1:800.
