# Supplementary material for: Leptospirosis occurs as frequently as malaria among adolescent and adult acute febrile patients in Hoima, Uganda: A prospective health facility-based study
Source: PLoS Negl Trop Dis. 2026 Oct 29;20(10):e0014772. doi: 10.1371/journal.pntd.0014772 (PMC13719026; doi:10.1371/journal.pntd.0014772)
Supplement: Supplementary file 8 [file pntd.0014772.s008.pdf]

S6 Table. Faine's Part A+B proxy performance

Diagnostic measure	Value (95% CI)
Sensitivity	4.5% (1.8-11.0)
Specificity	95.9% (92.5-97.7)
Positive predictive value	28.6% (11.7-54.6)
Negative predictive value	73.1% (68.0-77.7)

Applies the modified Faine's Part A+B items available on our case report form (headache, fever, fever ≥39°C, myalgia, abdominal pain or diarrhoea, jaundice, proteinuria or elevated creatinine, and any animal or water/flood contact) at the published cutoff of 26, tested against our conservative case definition (Bandara et al. 2016, BMC Infect Dis 16:446). Conjunctival suffusion and oliguria/anuria, both Part A components, were not captured on our case report form and are excluded from this proxy, so sensitivity here is a floor estimate rather than the true performance of the complete criteria. Jaundice occurred in only 1.1% of confirmed cases in this cohort, which limits how much higher the true value could plausibly be.
