# Supplementary material for: Leptospirosis occurs as frequently as malaria among adolescent and adult acute febrile patients in Hoima, Uganda: A prospective health facility-based study
Source: PLoS Negl Trop Dis. 2026 Oct 29;20(10):e0014772. doi: 10.1371/journal.pntd.0014772 (PMC13719026; doi:10.1371/journal.pntd.0014772)
Supplement: Supplementary file 9 [file pntd.0014772.s009.pdf]

**S7 Table. Discrimination by predictor set**

| Predictor set | Apparent AUC | 10-fold cross-validated AUC (SD) |
|---|---|---|
| Clinical features alone* | 0.58 | 0.48 (0.12) |
| Exposure history alone† | 0.64 | 0.63 (0.08) |
| Combined | 0.67 | 0.58 (0.10) |

**Headache, myalgia, abdominal pain, chills, jaundice, generalised tenderness. †Skinning, water or flood exposure. Cross-validated AUC is the mean of 10 held-out folds and is the more honest estimate of how each predictor set would perform on new patients; the apparent AUC is fit on the full sample and is optimistic.*
