# Supplementary material for: Leptospirosis occurs as frequently as malaria among adolescent and adult acute febrile patients in Hoima, Uganda: A prospective health facility-based study
Source: PLoS Negl Trop Dis. 2026 Oct 29;20(10):e0014772. doi: 10.1371/journal.pntd.0014772 (PMC13719026; doi:10.1371/journal.pntd.0014772)
Supplement: Supplementary file 10 [file pntd.0014772.s010.pdf]

S8 Table. Odds ratios, combined exploratory clinical-epidemiological model

Variable	OR	95% CI	p
Headache	1.08	0.48-2.43	0.85
Myalgia	0.96	0.53-1.74	0.89
Abdominal pain	0.81	0.47-1.41	0.45
Chills	1.09	0.63-1.89	0.76
Jaundice	1.24	0.35-4.42	0.74
Generalised tenderness	1.57	0.86-2.85	0.14
Skinning	3.09	0.95-10.03	0.061
Water or flood exposure	0.28	0.14-0.56	<0.001

Combined logistic model of all eight variables above, outcome is the conservative leptospirosis case definition. 89 cases and 8 predictors give 11.1 events per variable, at the low end of what is considered stable for a multivariable model; results are exploratory and hypothesis-generating, not a validated diagnostic tool. Reference: Rajapakse S et al. A diagnostic scoring model for leptospirosis in resource limited settings. PLoS Negl Trop Dis 2016; 10: e0004513, for comparison, a model incorporating laboratory markers (creatinine, bilirubin, platelets, neutrophil differential) in a Sri Lankan cohort achieved AUC 0.762, appreciably higher than the clinical/exposure-only discrimination found here.
